# Difficult-to-treat resistance (DTR), treatment, and outcomes of carbapenem-resistant Enterobacterales infections in the setting of IMP-type carbapenemase predominance in Japan

**DOI:** 10.1128/spectrum.01007-26

**Published:** 2026-04-27

**Authors:** Koji Ohyama, Kohei Uemura, Yasufumi Matsumura, Ryota Hase, Hideaki Kato, Takashi Matono, Naoya Itoh, Takehiro Hashimoto, Go Yamamoto, Momoko Mawatari, Takeya Tsutsumi, Tetsuya Suzuki, Masahiro Suzuki, Takuya Hosoda, Aki Sakurai, Yusuke Asai, Shinya Tsuzuki, Kayoko Hayakawa, David van Duin, Norio Ohmagari, Yohei Doi, Sho Saito

**Affiliations:** 1 Department of Microbiology, Fujita Health University School of Medicine 12695, Toyoake, Aichi, Japan; 2 Biostatistics and Bioinformatics Course, The University of Tokyo 13143, Bunkyo City, Tokyo, Japan; 3 Department of Clinical Laboratory Medicine, Kyoto University Graduate School of Medicine 593288 , Kyoto, Japan; 4 Department of Infectious Diseases, Japanese Red Cross Narita Hospital 13681, Narita, Chiba, Japan; 5 Infection Prevention and Control Department, Yokohama City University Hospital 218758, Yokohama, Kanagawa, Japan; 6 Division of Infectious Disease and Hospital Epidemiology, Saga University Hospital 469771, Saga, Japan; 7 Department of Infectious Diseases, Graduate School of Medical Sciences, Nagoya City University 12963, Nagoya, Aichi, Japan; 8 Hospital Infection Control Center, Oita University Hospital 105236, Yufu, Oita, Japan; 9 Department of Infection Control and Prevention, Osaka University Hospital 157453, Suita, Osaka, Japan; 10 Department of Transformative Infectious Disease Control Research, The University of Osaka Graduate School of Medicine, Suita, Osaka, Japan; 11 Department of Infectious Diseases, Japanese Red Cross Medical Center 26307, Shibuya, Tokyo, Japan; 12 Department of Infectious Diseases, The University of Tokyo Hospital 26782, Bunkyo City, Tokyo, Japan; 13 Department of Infection Control and Prevention, University of Yamanashi Hospital 231202, Chuo City, Yamanashi, Japan; 14 Disease Control and Prevention Center, National Center for Global Health and Medicine, Japan Institute for Health Security 739298 , Shinjuku City, Tokyo, Japan; 15 AMR Clinical Reference Center, National Center for Global Health and Medicine, Japan Institute for Health Security 739298 , Shinjuku City, Tokyo, Japan; 16 Faculty of Medicine and Health Sciences, University of Antwerp 26660, Antwerp, Belgium; 17 Division of Infectious Diseases, University of North Carolina School of Medicine 6797, Chapel Hill, North Carolina, USA; 18 Department of Infectious Diseases, Fujita Health University School of Medicine 89305, Toyoake, Aichi, Japan; 19 Division of Infectious Diseases, University of Pittsburgh School of Medicine 12317 , Pittsburgh, Pennsylvania, USA; University at Albany, Albany, New York, USA

**Keywords:** difficult-to-treat resistance, carbapenem-resistant, IMP carbapenemase, Enterobacterales, genome epidemiology, clinical outcome

## Abstract

Carbapenem-resistant Enterobacterales (CRE) pose a global threat due to limited treatment options and high mortality. Difficult-to-treat resistance (DTR), defined as non-susceptibility to all conventional β-lactams and fluoroquinolones, has primarily been applied to *Pseudomonas aeruginosa*. However, its relevance for Enterobacterales remains unclear, particularly in regions with a distinct carbapenemase landscape, such as Japan where IMP-type metallo-β-lactamases predominate. We analyzed the MultiDrug-Resistant organisms clinical research network (MDRnet) cohort, a multicenter prospective study conducted at 13 Japanese hospitals between April 2019 and March 2024. This analysis included patients with clinically indicated cultures yielding CRE. Clinical characteristics and outcomes assessed using the desirability of outcome ranking (DOOR) framework and genomic epidemiology characterized by whole-genome sequencing were compared between DTR and non-DTR groups. Among 196 CRE cases, 64 (32.7%) represented infections, including 12 DTR cases (18.8%). Carbapenemase genes were detected in 34/64 infections (53.1%), with similar prevalence in the DTR and non-DTR groups (50.0% vs 53.8%). *bla*_IMP-1_ was the most frequently identified carbapenemase gene (*n* = 28). The overall 30-day mortality rate was 21.9%, with 16.7% (95% confidence interval [CI], 2.1%–48.4%) in the DTR group and 23.1% (95% CI, 12.5%–36.8%) in the non-DTR group. DOOR outcomes were similar between groups. Appropriate empiric and definitive therapy was less frequently administered in the DTR group. In this cohort, where IMP-type carbapenemases and non-DTR CRE are prevalent, DTR classification did not appear to correlate with 30-day mortality or DOOR outcomes. These findings underscore the importance of regional molecular epidemiology when interpreting clinical outcomes of CRE infections.

**IMPORTANCE:**

Difficult-to-treat resistance (DTR) is increasingly used to assess the clinical impact of antimicrobial resistance, but its significance in carbapenem-resistant Enterobacterales (CRE) may vary across molecular epidemiologic settings. In this prospective multicenter study from Japan, where IMP-type carbapenemases predominated, most CRE infections were classified as non-DTR. Whole-genome sequencing also revealed a distinct local clonal structure. DTR was not associated with 30-day mortality or desirability of outcome ranking despite lower rates of appropriate empiric and definitive therapy. Our study highlights the importance of interpreting DTR in CRE within the context of regional molecular epidemiology and supports the need for continued regional multicenter studies to evaluate its clinical utility.

## INTRODUCTION

Carbapenem-resistant Enterobacterales (CRE) are a major global health threat due to their resistance to nearly all conventional antibiotics including carbapenems, which are often reserved as last-resort agents for severe infections (1). This resistance renders CRE infections particularly difficult to treat and is associated with high mortality rates, reaching up to 50% in cases such as bloodstream infections or pneumonia (2–4). The continued spread of CRE poses a major concern because of the limited availability of effective treatment options.

In several East Asian countries, including Japan, IMP-type metallo-β-lactamases are the predominant carbapenemases detected among CRE isolates (5). In contrast to CRE producing globally common carbapenemases such as KPC, NDM, and VIM, IMP-producing strains may remain susceptible to agents including piperacillin-tazobactam, aztreonam, and occasionally cefepime and fluoroquinolones (6, 7). Reflecting this broader susceptibility profile, infections caused by IMP-producing CRE have not consistently been associated with worse outcomes compared to those caused by carbapenem-susceptible strains, possibly due to the retained clinical efficacy of certain non-carbapenem agents (8, 9).

The concept of difficult-to-treat resistance (DTR) among Gram-negative bacteria has emerged in response to the increasing prevalence of multidrug resistance. DTR is defined by non-susceptibility to all conventional β-lactam agents, including β-lactam/β-lactamase inhibitor (BL/BLI) combinations, expanded-spectrum cephalosporins, and carbapenems, as well as to fluoroquinolones (10). By this definition, DTR organisms are a subset of carbapenem-resistant strains that exhibit broad resistance beyond carbapenems, leaving clinicians with few, if any, safe and effective treatment options for patients.

Although the DTR classification was initially proposed for all major Gram-negative pathogens including Enterobacterales, *Pseudomonas aeruginosa,* and *Acinetobacter baumannii*, it has been predominantly applied to *P. aeruginosa* (10, 11). This is partly because carbapenem-resistant *P. aeruginosa* may retain susceptibility to other β-lactam or fluoroquinolone agents with antipseudomonal activity, making the DTR classification clinically useful for guiding appropriate therapy (12). In contrast, CRE isolates, particularly those producing globally common carbapenemases such as KPC, NDM, or VIM, typically exhibit broader resistance, often including other β-lactams and fluoroquinolones (13, 14). Accordingly, treatment strategies for CRE tend to focus more on the identification and characterization of specific carbapenemase enzymes (e.g., serine carbapenemases like KPC or metallo-β-lactamases like NDM and VIM), rather than phenotypic classifications such as DTR (15, 16).

Given the distinct resistance landscape of IMP-predominant settings, we hypothesized that the DTR classification may provide clinical utility in stratifying CRE-infected patients who are at higher risk for poor outcomes. We therefore evaluated the clinical utility of the DTR classification by analyzing patient outcomes in terms of mortality and the desirability of outcome ranking (DOOR) using data from a multicenter, prospective observational study that was conducted to characterize the molecular and clinical epidemiology of CRE in Japan.

## MATERIALS AND METHODS

### Study design and patients

This analysis was conducted using data from the MultiDrug-Resistant organisms clinical research network (MDRnet), a multicenter, prospective observational study conducted across 13 tertiary care hospitals in Japan. The MDRnet cohort aimed to elucidate the epidemiology of carbapenem-resistant Gram-negative bacteria. Between April 2019 and March 2024, hospitalized patients with a clinically indicated culture yielding CRE were prospectively identified and enrolled (17). Susceptibility testing and whole-genome sequencing (WGS) were performed at the MDRnet central laboratory at Fujita Health University. Isolates were included if they were non-susceptible to meropenem by disk diffusion (zone diameter, ≤22 mm) according to the Clinical and Laboratory Standards Institute (CLSI) criteria (M100, 31st edition) (18), or if they were judged to be carbapenemase-producing Enterobacterales at the participating institution according to local laboratory practice. This approach was used to capture carbapenemase-producing Enterobacterales with relatively low carbapenem minimum inhibitory concentrations (MICs) or larger inhibition zones that might otherwise be missed by phenotype-based screening alone. WGS was performed subsequently as part of the centralized study analysis. For the present analysis, only patients with confirmed CRE infections were included, and cases of colonization without evidence of infection were excluded. Additionally, only the first infection episode caused by CRE during each hospitalization was analyzed.

### Antimicrobial susceptibility testing

MICs were determined using the broth microdilution method with custom-made dry plates (Eiken Chemical Co., Tokyo, Japan). Results were interpreted according to the CLSI criteria (M100, 31st edition) (18). Additional susceptibility testing for cefiderocol was performed by broth microdilution using a commercially prepared dry plate (Kyokuto Pharmaceutical Industrial Co., Tokyo, Japan), and results were interpreted according to the CLSI criteria (M100, 34th edition) (19). DTR was defined as non-susceptibility to all conventional β-lactams and fluoroquinolones following established criteria (10, 20). The fluoroquinolones tested included ciprofloxacin and levofloxacin. The β-lactam category encompassed carbapenems (imipenem, meropenem), an extended-spectrum cephalosporin (cefepime), a BL/BLI combination (piperacillin-tazobactam), and a monobactam (aztreonam).

### Whole-genome sequencing

Genomic DNA was extracted from isolates using the Puregene Cell Kit (Qiagen, Tokyo, Japan) according to the manufacturer’s instructions. DNA libraries were prepared using the QIAseq FX DNA Library Kit (Qiagen, Tokyo, Japan), and WGS was performed using the NextSeq 2000 platform (Illumina, Inc., San Diego, CA, USA), with 2 × 150 bp paired-end reads. *De novo* genome assembly was conducted using SPAdes v3.13.1 (21). Carbapenemase production was inferred based on the presence of carbapenemase genes, identified via AMRFinderPlus v4.0.19 (22).

Species identification was conducted using orthologous average nucleotide identity (OrthoANI) v1.2, with 95% and 98% cutoffs applied to define species and subspecies, respectively, based on whole-genome comparisons with reference strains (23, 24). Multilocus sequence typing was performed *in silico* using mlst v2.18.0, available at the software repository (https://github.com/tseemann/mlst). For isolates lacking identifiable carbapenemase genes, the modified carbapenemase inactivation method was used to rule out carbapenemase production (25).

### Definitions of infection

The classification of infection vs colonization followed previously established criteria (26). A patient was deemed to have an infection if CRE was isolated from blood or other sterile body sites. For bloodstream infection, pneumonia, urinary tract infection, surgical site infection, and intra-abdominal infection, the Centers for Disease Control and Prevention surveillance definitions were applied (27–31).

### Data collection

Clinical data were extracted from medical records and entered into a REDCap database. Collected variables included demographics, clinical characteristics, antimicrobial treatments, and clinical outcomes. Empiric therapy was defined as any antimicrobial agent with Gram-negative activity administered at least once within 3 days following collection of the index culture. Definitive therapy was defined as antimicrobial treatment initiated more than 3 days after culture collection and continued for a minimum of 2 days (26). Antimicrobials initiated prior to the availability of susceptibility results were also considered definitive if continued thereafter. Therapy was considered appropriate if the pathogen was susceptible to the agents based on centrally conducted MIC results interpreted according to CLSI M100 (31st edition).

The DOOR was evaluated using three components: clinical response, infectious complications, and serious adverse events, as detailed in Table S1 (32–34). The most favorable outcome was defined as survival without any component events, and the worst outcome was death.

### Statistical analysis

Comparative analyses were conducted between the DTR and non-DTR groups. Continuous variables were summarized as medians with interquartile ranges (IQRs), while categorical variables were reported as frequencies and percentages. Wilcoxon’s rank sum test was used for continuous variables, and Fisher’s exact test was used for categorical variables. Exact binomial 95% confidence intervals (CIs) were calculated for proportions, as appropriate. Thirty-day mortality was analyzed using Kaplan-Meier curves and the log-rank test. DOOR analyses were performed to assess overall clinical outcomes using a web-based application (35). For the DOOR analysis, a probability of 50% indicates no difference between groups. A 95% CI including 50% was interpreted as not statistically significant (32, 34). All tests were two-sided, and *P* values <0.05 were considered statistically significant. All statistical analyses were conducted using Stata software, v18.5.

## RESULTS

### Patient characteristics

A total of 196 patients with CRE isolates were enrolled from 13 hospitals across Japan. Among them, 64 (32.7%) had confirmed CRE infections.

Baseline characteristics of patients with CRE infections are summarized in Table 1. The cohort was predominantly elderly, with a median age of 71 years (IQR, 62.5–77.0), and the majority were male (71.9%). Most patients had chronic comorbidities, reflected by a median Charlson comorbidity index of 2 (IQR, 1–4), and 31.2% had immunosuppression. Hospital-acquired infections accounted for 82.8% of cases, followed by healthcare-associated (14.1%) and community-associated (3.1%) infections. Antimicrobial exposure within 14 days prior to culture collection was reported in 82.8% of patients. The median pre-culture length of hospital stay was 28.0 days (IQR, 8.0–45.0), and the median Pitt bacteremia score was 1 (IQR, 0–3).

**TABLE 1:** Baseline characteristics of patients with CRE infections^*a*^^,*b*,*c*^

	All (*n* = 64)	DTR CRE (*n* = 12)	Non-DTR CRE (*n* = 52)	IMP-producing CRE (*n* = 30)
Age, years	71.0 (62.5–77.0)	66.0 (60.5–75.5)	72.0 (62.5–77.5)	72.0 (63.0–75.0)
Male sex	46 (71.9)	9 (75.0)	37 (71.2)	20 (66.7)
Immunosuppression	20 (31.2)	4 (33.3)	16 (30.8)	8 (26.7)
Corticosteroid therapy	13 (20.3)	3 (25.0)	10 (19.2)	8 (26.7)
Chemotherapy	4 (6.2)	1 (8.3)	3 (5.8)	1 (3.3)
Biological agents	3 (4.7)	1 (8.3)	2 (3.8)	1 (3.3)
Immunosuppressive drugs	7 (10.9)	1 (8.3)	6 (11.5)	2 (6.7)
Solid organ transplantation	7 (10.9)	2 (16.7)	5 (9.6)	2 (6.7)
Hematopoietic stem cell transplantation	1 (1.6)	0 (0.0)	1 (1.9)	0 (0.0)
HIV infection	1 (1.6)	0 (0.0)	1 (1.9)	0 (0.0)
Charlson comorbidity index	2.0 (1.0–4.0)	2.0 (1.0–3.0)	2.5 (1.0–4.0)	2.0 (1.0–3.0)
Onset				
Community-associated infection	2 (3.1)	1 (8.3)	1 (1.9)	1 (3.3)
Healthcare-associated infection	9 (14.1)	0 (0.0)	9 (17.3)	5 (16.7)
Hospital-acquired infection	53 (82.8)	11 (91.7)	42 (80.8)	24 (80.0)
Recent healthcare exposures				
Hemodialysis	6 (9.4)	2 (16.7)	4 (7.7)	2 (6.7)
Hospitalization within 30 days	27 (42.2)	2 (16.7)	25 (48.1)	9 (30.0)
ICU admission within 30 days	18 (28.1)	5 (41.7)	13 (25.0)	8 (26.7)
Intravenous therapy within 30 days	54 (84.4)	10 (83.3)	44 (84.6)	25 (83.3)
Endoscopic procedure within 30 days	14 (21.9)	3 (25.0)	11 (21.2)	5 (16.7)
Surgery within 30 days	18 (28.1)	2 (16.7)	16 (30.8)	12 (40.0)
Antibiotic exposure within 14 days	53 (82.8)	10 (83.3)	43 (82.7)	23 (76.7)
Pre-culture length of hospital stay, days	28.0 (8.0–45.0)	30.0 (11.0–47.0)	28.0 (8.0–45.0)	27.5 (7.0–41.0)
Pitt bacteremia score	1.0 (0.0–3.0)	1.5 (0.0–5.0)	1.0 (0.0–3.0)	1.0 (0.0–3.0)
Infection source				
Bloodstream infection	16 (25.0)	1 (8.3)	15 (28.8)	9 (30.0)
Pneumonia	8 (12.5)	3 (25.0)	5 (9.6)	4 (13.3)
Urinary tract infection	17 (26.6)	3 (25.0)	14 (26.9)	9 (30.0)
Surgical site infection	9 (14.1)	2 (16.7)	7 (13.5)	4 (13.3)
Intra-abdominal infection	13 (20.3)	3 (25.0)	10 (19.2)	4 (13.3)
Vascular-graft infection	1 (1.6)	0 (0.0)	1 (1.9)	0 (0.0)

^*a*^ Data are *n* (%) or median (IQR).

^*b*^ No statistically significant differences were observed between the DTR and non-DTR CRE groups. The IMP-producing column is descriptive.

^*c*^ CRE, carbapenem-resistant Enterobacterales; DTR, difficult-to-treat resistance; ICU, intensive care unit.

Regarding infection sites, urinary tract infections were the most common (26.6%), followed by bloodstream infections (25.0%) and intra-abdominal infections (20.3%). There were no significant differences in patient demographics between the DTR and non-DTR groups. Baseline characteristics in the IMP-producing subset were broadly similar to those in the overall cohort (Table 1).

### Antimicrobial treatment

Among the 64 infection cases, 12 (18.8%) were classified as DTR CRE infections, while 52 (81.2%) were classified as non-DTR CRE infections. Carbapenems and BL/BLI combinations, predominantly piperacillin-tazobactam, were the most frequently administered empiric agents in both groups. Overall, only 25.0% of patients received appropriate empiric therapy, including 8.3% in the DTR group and 28.8% in the non-DTR group (Table 2).

**TABLE 2:** Empiric and definitive antimicrobial therapy^*a*^^,*b*,*c*^

	All (*n* = 64)	DTR CRE (*n* = 12)	Non-DTR CRE (*n* = 52)	IMP-producing CRE (*n* = 30)	*P* value
Empiric therapy					
Appropriate	16 (25.0)	1 (8.3)	15 (28.8)	6 (20.0)	0.266
Combination	8 (12.5)	1 (8.3)	7 (13.5)	3 (10.0)	1.000
Aztreonam	1 (1.6)	0 (0.0)	1 (1.9)	1 (3.3)	1.000
BL/BLI	25 (39.1)	3 (25.0)	22 (42.3)	13 (43.3)	0.338
Carbapenem	23 (35.9)	4 (33.3)	19 (36.5)	6 (20.0)	1.000
Extended-spectrum cephalosporin	14 (21.9)	3 (25.0)	11 (21.2)	9 (30.0)	0.715
Fluoroquinolone	7 (10.9)	0 (0.0)	7 (13.5)	4 (13.3)	0.331
Aminoglycoside	2 (3.1)	0 (0.0)	2 (3.8)	1 (3.3)	1.000
Others	6 (9.4)	1 (8.3)	5 (9.6)	3 (10.0)	1.000
Definitive therapy					
Appropriate	31 (48.4)	2 (16.7)	29 (55.8)	17 (56.7)	0.023
Combination	17 (26.6)	3 (25.0)	14 (26.9)	10 (33.3)	1.000
Aztreonam	8 (12.5)	1 (8.3)	7 (13.5)	6 (20.0)	1.000
BL/BLI	17 (26.6)	4 (33.3)	13 (25.0)	9 (30.0)	0.718
Carbapenem	20 (31.2)	3 (25.0)	17 (32.7)	8 (26.7)	0.739
Extended-spectrum cephalosporin	14 (21.9)	2 (16.7)	12 (23.1)	6 (20.0)	1.000
Fluoroquinolone	18 (28.1)	3 (25.0)	15 (28.8)	12 (40.0)	1.000
Aminoglycoside	10 (15.6)	1 (8.3)	9 (17.3)	7 (23.3)	0.672
Others	10 (15.6)	1 (8.3)	9 (17.3)	3 (10.0)	0.672
Total duration of therapy	15.0 (8.5–27.5)	11.0 (6.5–19.5)	16.0 (9.5–28.0)	14.0 (7.0–22.0)	0.252

^*a*^ Data are *n* (%) or median (IQR).

^*b*^ *P* values compare DTR vs non-DTR groups. The IMP-producing column is descriptive. Treatment regimens may have included more than one of the listed antibiotics in some patients. Other antibiotics included cefmetazole, flomoxef, minocycline, piperacillin, trimethoprim-sulfamethoxazole, and tigecycline.

^*c*^ BL/BLI, β-lactam/β-lactamase inhibitor; CRE, carbapenem-resistant Enterobacterales; DTR, difficult-to-treat resistance.

For definitive therapy among all patients, carbapenems, fluoroquinolones, and BL/BLI combinations (primarily piperacillin-tazobactam) were the most commonly prescribed agents. Combination therapy was administered to 26.6% of all patients, often involving a β-lactam (e.g., aztreonam or a carbapenem) combined with a fluoroquinolone or an aminoglycoside. Trimethoprim-sulfamethoxazole was used in only two cases (3.1%), and tigecycline was used in one case (1.6%). Appropriate definitive therapy was given to 48.4% of all patients: 16.7% in the DTR group and 55.8% in the non-DTR group. In the IMP-producing subset, treatment patterns were generally similar to those in the non-DTR group, although fluoroquinolones, aminoglycosides, and aztreonam were used somewhat more frequently in definitive therapy (Table 2).

### Genomic epidemiology

WGS revealed the presence of carbapenemase genes in 34 of 64 isolates (53.1%), with comparable prevalence between the DTR and non-DTR groups (50.0% vs 53.8%) (Table 3). The most common *bla*_IMP_ gene was *bla*_IMP-1_ (*n* = 28), followed by *bla*_IMP-11_ and *bla*_IMP-60_ (*n* = 1 each). Among non-*bla*_IMP_ carbapenemase genes, *bla*_GES-5_ was detected in three isolates and *bla*_NDM-1_ in one.

**TABLE 3:** Bacterial characteristics including species distribution and carbapenemase genes^*a*^^,*b*^

	All (*n* = 64)	DTR CRE (*n* = 12)	Non-DTR CRE (*n* = 52)	*P* value^*c*^
Species				0.239
*Enterobacter cloacae* complex	21 (32.8)	4 (33.3)	17 (32.7)	
*Enterobacter hormaechei* subsp*. steigerwaltii*	11 (17.2)	2 (16.7)	9 (17.3)	
*Enterobacter hormaechei* subsp*. hoffmannii*	3 (4.7)	2 (16.7)	1 (1.9)	
*Enterobacter hormaechei* subsp*. xiangfangensis*	1 (1.6)	0 (0.0)	1 (1.9)	
*Enterobacter asburiae*	4 (6.2)	0 (0.0)	4 (7.7)	
*Enterobacter kobei*	1 (1.6)	0 (0.0)	1 (1.9)	
*Enterobacter quasiroggenkampii*	1 (1.6)	0 (0.0)	1 (1.9)	
*Klebsiella aerogenes*	8 (12.5)	1 (8.3)	7 (13.5)	
*Klebsiella pneumoniae* complex	13 (20.3)	2 (16.7)	11 (21.2)	
*Klebsiella pneumoniae* subsp. *pneumoniae*	11 (17.2)	2 (16.7)	9 (17.3)	
*Klebsiella quasipneumoniae* subsp*. similipneumoniae*	1 (1.6)	0 (0.0)	1 (1.9)	
*Klebsiella variicola*	1 (1.6)	0 (0.0)	1 (1.9)	
*Klebsiella oxytoca* complex	8 (12.5)	1 (8.3)	7 (13.5)	
*Klebsiella oxytoca*	1 (1.6)	1 (8.3)	0 (0.0)	
*Klebsiella michiganensis*	7 (10.9)	0 (0.0)	7 (13.5)	
*Escherichia coli*	3 (4.7)	1 (8.3)	2 (3.8)	
*Citrobacter* spp.	4 (6.2)	0 (0.0)	4 (7.7)	
*Citrobacter braakii*	1 (1.6)	0 (0.0)	1 (1.9)	
*Citrobacter europaeus*	1 (1.6)	0 (0.0)	1 (1.9)	
*Citrobacter freundii*	2 (3.1)	0 (0.0)	2 (3.8)	
*Proteus mirabilis*	3 (4.7)	0 (0.0)	3 (5.8)	
*Serratia* spp.	4 (6.2)	3 (25.0)	1 (1.9)	
*Serratia nevei*	3 (4.7)	2 (16.7)	1 (1.9)	
*Serratia sarumanii*	1 (1.6)	1 (8.3)	0 (0.0)	
Carbapenemases	34 (53.1)	6 (50.0)	28 (53.8)	1.000
*bla*_IMP-1_	28 (43.8)	4 (33.3)	24 (46.2)	
*bla*_IMP-11_	1 (1.6)	0 (0.0)	1 (1.9)	
*bla*_IMP-60_	1 (1.6)	0 (0.0)	1 (1.9)	
*bla*_NDM-1_	1 (1.6)	0 (0.0)	1 (1.9)	
*bla*_GES-5_	3 (4.7)	2 (16.7)	1 (1.9)	

^*a*^ Data are *n* (%).

^*b*^ CRE, carbapenem-resistant Enterobacterales; DTR, difficult-to-treat resistance.

^*c*^ *P* values compare the overall distribution of species and carbapenemase categories between the DTR and non-DTR groups.

The most frequently identified species were *Enterobacter hormaechei* subsp. *steigerwaltii* (*n* = 11, 17.2%) and *Klebsiella pneumoniae* subsp. *pneumoniae* (*n* = 11, 17.2%), followed by *Klebsiella aerogenes* (*n* = 8, 12.5%). The most prevalent sequence type (ST) was ST133 of *E. hormaechei* subsp. *steigerwaltii* (*n* = 7, 10.9%), followed by ST78 of *E. hormaechei* subsp. *hoffmannii* (*n* = 3, 4.7%) and ST43 of *Klebsiella michiganensis* (*n* = 3, 4.7%) (Table S2).

All *bla*_GES-5_-carrying isolates (*n* = 3) were *Serratia nevei*, with two belonging to the DTR group. The single *bla*_NDM-1_-harboring isolate, found in *E. hormaechei* subsp. *steigerwaltii*, was classified as non-DTR. *Serratia* spp. (*S. nevei* [*n* = 3] and *S. sarumanii* [*n* = 1]) accounted for 25.0% of DTR isolates and 1.9% of non-DTR isolates. *E. hormaechei* subsp. *hoffmannii* accounted for 16.7% of DTR isolates and 1.9% of non-DTR isolates.

### Antimicrobial susceptibility

Overall, aminoglycosides retained high activity in this cohort, with susceptibility rates of 98.4% for amikacin, 93.8% for gentamicin, and 78.1% for tobramycin. Tigecycline (95.3%) and trimethoprim-sulfamethoxazole (71.9%) also showed activity (Table 4). Cefiderocol likewise showed high activity, with 90.6% of isolates categorized as susceptible overall, including 100% of DTR isolates and 88.5% of non-DTR isolates. Because DTR isolates are, by definition, non-susceptible to all tested conventional β-lactams and fluoroquinolones, susceptibility rates for these agents are reported for the non-DTR group only. Among non-DTR isolates, aztreonam showed the highest susceptibility among β-lactams (35.9%), followed by imipenem (28.1%) and meropenem (25.0%). Susceptibility to ciprofloxacin and levofloxacin was 40.6% and 37.5%, respectively.

**TABLE 4:** Antimicrobial susceptibility profiles^*a*^^,*b*,*c*^

Antibiotic	All (*n* = 64)	DTR CRE (*n* = 12)	Non-DTR CRE (*n* = 52)	IMP-producing CRE (*n* = 30)
Meropenem	16 (25.0)	0 (0.0)	16 (30.8)	4 (13.3)
Imipenem	18 (28.1)	0 (0.0)	18 (34.6)	6 (20.0)
Cefepime	11 (17.2)	0 (0.0)	11 (21.2)	1 (3.3)
Aztreonam	23 (35.9)	0 (0.0)	23 (44.2)	17 (56.7)
Piperacillin-tazobactam	12 (18.8)	0 (0.0)	12 (23.1)	10 (33.3)
Ciprofloxacin	26 (40.6)	0 (0.0)	26 (50.0)	11 (36.7)
Levofloxacin	24 (37.5)	0 (0.0)	24 (46.2)	10 (33.3)
Amikacin	63 (98.4)	12 (100.0)	51 (98.1)	30 (100.0)
Gentamicin	60 (93.8)	10 (83.3)	50 (96.2)	30 (100.0)
Tobramycin	50 (78.1)	8 (66.7)	42 (80.8)	23 (76.7)
Fosfomycin	38 (59.4)	5 (41.7)	33 (63.5)	19 (63.3)
Colistin	47 (73.4)	7 (58.3)	40 (76.9)	25 (83.3)
Tigecycline	61 (95.3)	12 (100.0)	49 (94.2)	29 (96.7)
Minocycline	26 (40.6)	4 (33.3)	22 (42.3)	11 (36.7)
Trimethoprim-sulfamethoxazole	46 (71.9)	10 (83.3)	36 (69.2)	24 (80.0)
Cefiderocol	58 (90.6)	12 (100.0)	46 (88.5)	30 (100.0)

^*a*^ Data are *n* (%).

^*b*^ Isolates for which susceptibility results were unavailable were classified as non-susceptible. For colistin, CLSI defines no susceptible category; MIC ≤ 2 μg/mL is considered intermediate and was reported as susceptible for consistency. For tigecycline, CLSI breakpoints are not defined; therefore, the FDA criterion for susceptibility (MIC ≤ 2 μg/mL) was applied.

^*c*^ CRE, carbapenem-resistant Enterobacterales; DTR, difficult-to-treat resistance.

In the IMP-producing subset, aminoglycosides also retained high activity, consistent with the overall cohort. Cefiderocol also showed high activity in this subset, with all isolates categorized as susceptible. Notably, aztreonam showed the highest susceptibility among β-lactams (56.7%), whereas susceptibility to fluoroquinolones was limited (Table 4).

### Clinical outcomes

**Fig 1:**
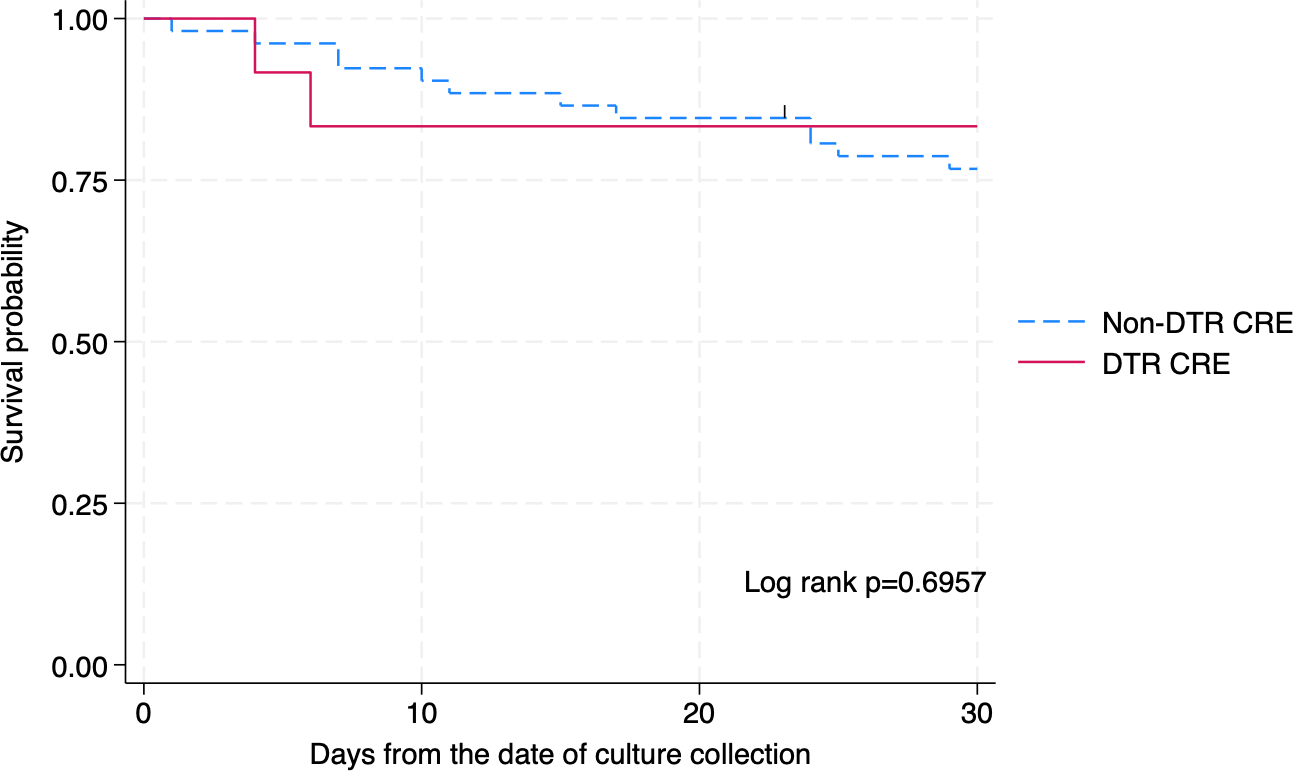
Kaplan-Meier survival curve comparing 30-day mortality between patients with DTR CRE and non-DTR CRE infections. CRE, carbapenem-resistant Enterobacterales; DTR, difficult-to-treat resistance.

The overall 30-day mortality rate was 21.9%, with 16.7% (95% CI, 2.1%–48.4%) in the DTR group and 23.1% (95% CI, 12.5%–36.8%) in the non-DTR group. The risk ratio for 30-day mortality was 0.72 (95% CI, 0.19–2.81), with no statistically significant difference between groups (Table 5). Kaplan-Meier analysis (Fig. 1) showed no statistically significant difference in survival over 30 days (log-rank test, *P* = 0.6957), although DTR patients tended to die earlier in the clinical course. The two fatal cases in the DTR group involved pneumonia and intra-abdominal infection, respectively.

**TABLE 5:** Outcomes of patients with CRE infections^*a*^^,*b*,*c*^

	All (*n* = 64)	DTR CRE (*n* = 12)	Non-DTR CRE (*n* = 52)	IMP-producing CRE (*n* = 30)	*P* value
Patient outcomes					
30-day mortality	14 (21.9)	2 (16.7)	12 (23.1)	5 (16.7)	1.000
Total length of hospital stay, days	58.5 (32.5–110.0)	59.0 (35.5–104.5)	58.5 (29.5–110.0)	67.0 (26.0–109.0)	0.783
Hospital stay after culture collection, days	26.0 (14.0–66.5)	28.5 (10.5–52.5)	25.0 (14.5–75.5)	34.5 (11.0–74.0)	0.600
Readmission within 90 days	14 (21.9)	1 (8.3)	13 (25.0)	6 (20.0)	0.267
Disposition					0.764
Death	22 (34.4)	3 (25.0)	19 (36.5)	8 (26.7)	
Transfer to another hospital	14 (21.9)	3 (25.0)	11 (21.2)	7 (23.3)	
Home discharge	27 (42.2)	6 (50.0)	21 (40.4)	14 (46.7)	
Transfer to a long-term care facility	1 (1.6)	0 (0.0)	1 (1.9)	1 (3.3)	

^*a*^ Data are *n* (%) or median (IQR).

^*b*^ *P* values compare the DTR and non-DTR groups. The IMP-producing CRE column is descriptive. One patient in the non-DTR group was discharged alive on day 23 after culture collection, and 30-day mortality was classified as unknown because follow-up data were unavailable, according to the study definitions.

^*c*^ CRE, carbapenem-resistant Enterobacterales; DTR, difficult-to-treat resistance.

**Fig 2:**
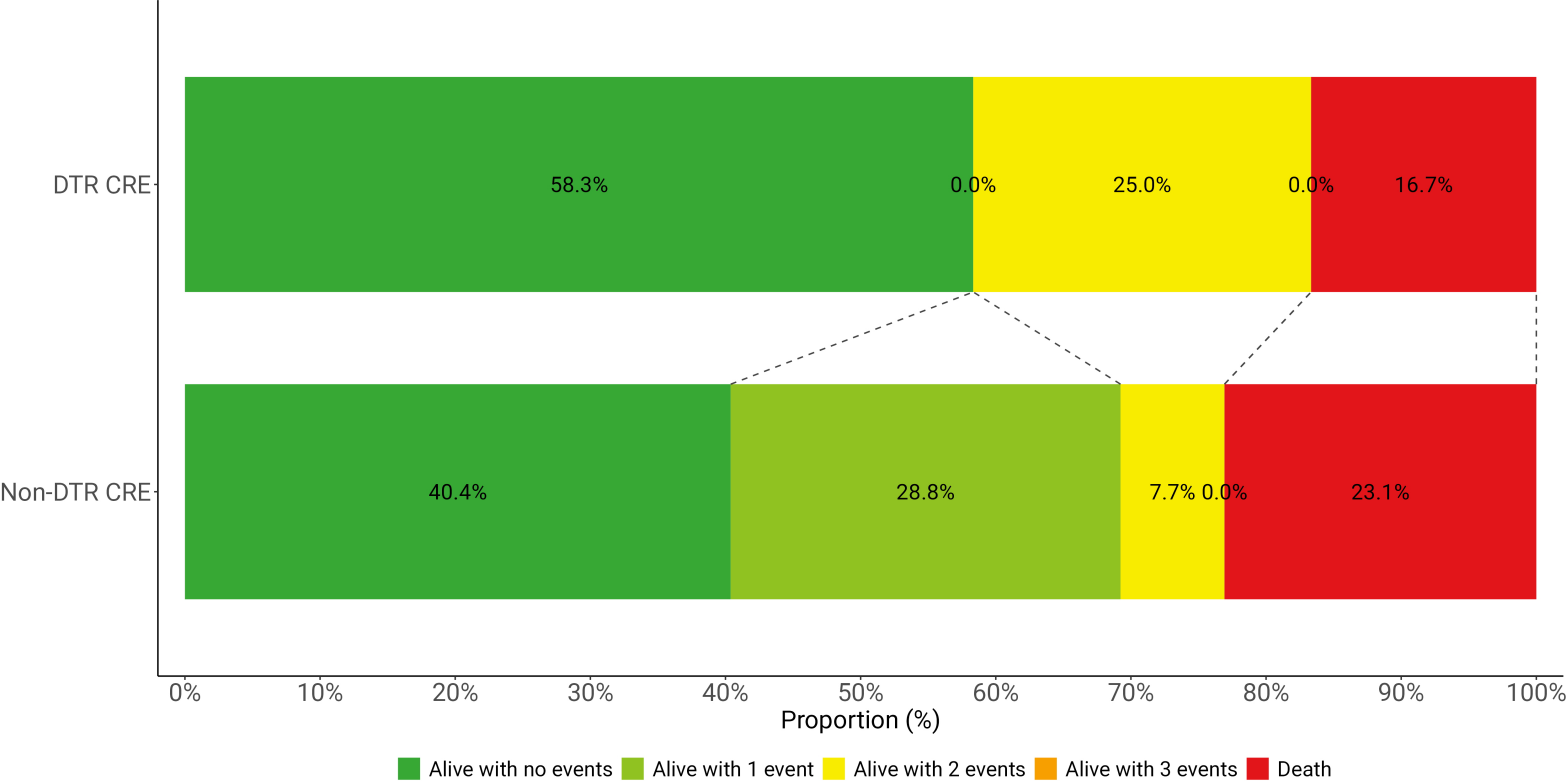
Desirability of outcome ranking (DOOR) categories in patients with DTR and non-DTR CRE infections. CRE, carbapenem-resistant Enterobacterales; DTR, difficult-to-treat resistance.

DOOR results were similar between groups. The probability of a more desirable outcome in the DTR group than in the non-DTR group was 55.2% (95% CI, 37.3%–71.9%) (Fig. 2), indicating no statistically significant difference with the 95% CI including 50%. In addition, no significant differences were observed in any of the undesirable event components of the DOOR ranking between the DTR and non-DTR groups (Fig. S1).

The median length of hospitalization was 58.5 days (IQR, 32.5–110.0), and the median length of hospital stay after culture collection was 26.0 days (IQR, 14.0–66.5). Upon discharge, 34.4% of patients had died, 21.9% were transferred to other hospitals, 42.2% were discharged home, and 1.6% were discharged to long-term care facilities. There were no significant differences between the DTR and non-DTR groups for these outcomes. In the IMP-producing subset, clinical outcomes were similar to those in the overall cohort (Table 5).

## DISCUSSION

This multicenter study provides important insights into the clinical and microbiological profiles of DTR and non-DTR CRE infections in Japan. Approximately 19% of CRE infections met the criteria for DTR, while the remaining 81% were classified as non-DTR. This predominance of non-DTR CRE contrasts with data from regions where globally common carbapenemase genes such as *bla*_KPC_, *bla*_NDM_, and *bla*_VIM_ are prevalent and where the proportions of DTR and carbapenem resistance tend to be more closely aligned (10, 12). The high proportion of non-DTR CRE observed in our cohort reflects the regional predominance of CRE isolates producing IMP-type metallo-β-lactamases, which are known to confer a narrower spectrum of resistance and often preserve susceptibility to some β-lactams and fluoroquinolones (5–7).

Prior studies have linked DTR Gram-negative infections to inappropriate or delayed therapy and limited treatment options, which have been associated with higher mortality, particularly for *P. aeruginosa* (36, 37). However, among Enterobacterales, in settings where globally prevalent carbapenemases predominate, resistance to carbapenems frequently overlaps with DTR, making it difficult to distinguish the prognostic impact of DTR alone (10–12, 38). In settings such as Japan, where IMP-type carbapenemases predominate and DTR is not closely aligned with carbapenem resistance, we hypothesized that the DTR classification might help identify high-risk patients.

Contrary to our expectation, our analysis did not support the hypothesis that 30-day mortality or DOOR outcomes differ between patients with DTR CRE and those with non-DTR CRE infections. This was observed despite a lower frequency of appropriate empiric and definitive therapy in the DTR group. During the study period, rates of appropriate therapy were low overall in both groups. This finding should be interpreted in the context of limited access during the study period to newer agents with reliable activity against CRE in Japan, including cefiderocol and newer BL/BLI combinations. In addition, the optimal therapy for infections caused by IMP-producing CRE has yet to be defined and may vary across settings (7, 39). Current guidance for MBL-producing Enterobacterales highlights ceftazidime-avibactam plus aztreonam and cefiderocol as preferred options (15). However, no patients received cefiderocol or ceftazidime-avibactam plus aztreonam during the study period. In our cohort, definitive regimens most commonly included carbapenems, piperacillin-tazobactam, and fluoroquinolones. Use of alternative agents was limited, and although cefiderocol susceptibility testing performed for this study demonstrated good *in vitro* activity, these results were not available to guide clinical decision-making during the study period.

Several factors may further explain the limited prognostic discrimination by DTR status in our cohort. The number of DTR cases was small (*n* = 12), limiting statistical power. In addition, our cohort comprised severely ill patients, with more than 80% hospital-acquired infections, a median of 28 days from admission to the first positive culture, and an in-hospital mortality exceeding 30%. The overall 30-day mortality was approximately 22%, which is higher than the 12%–15% reported in comparable epidemiologic settings (8, 9, 40). This high-acuity patient population suggests that outcomes may be influenced by multiple determinants beyond the antimicrobial resistance phenotype alone, potentially limiting prognostic discrimination by DTR status. Bloodstream infections were more frequent in the non-DTR group than in the DTR group, which may have further attenuated potential between-group differences in mortality.

WGS revealed frequent detection of several notable STs among CRE isolates, including ST133 of *E. hormaechei* subsp. *steigerwaltii*, ST78 of *E. hormaechei* subsp. *hoffmannii*, and ST43 of *K. michiganensis*. ST133 has been identified as a globally disseminated, carbapenem-resistant, and potentially invasive lineage of *Enterobacter cloacae* complex, underscoring its clinical importance (41, 42). ST78 is another well-recognized high-risk clone within the *E. cloacae* complex that is commonly linked to multidrug resistance (43, 44). Within the *Klebsiella oxytoca* complex, *K. michiganensis*, particularly ST43, has been implicated in regional outbreaks involving carbapenemase production in East Asia, including Japan and China (45–47).

Species-specific patterns were also observed. Although the number of isolates was small, *Serratia* spp. (*S. nevei* and *S. sarumanii*) and *E. hormaechei* subsp. *hoffmannii* were identified in both groups, with numerically different distributions between the DTR and non-DTR groups. Consistent with this observation, carbapenem-resistant *Serratia* spp. have been reported to exhibit broad resistance to β-lactams and fluoroquinolones (48). In our cohort, *Serratia* spp. isolates predominantly produced GES-type carbapenemases. In Japan, GES-producing *Serratia* spp. have been implicated in nosocomial outbreaks and have also been reported in other regions, underscoring the need to consider potential environmental reservoirs (49, 50). In addition, ST78 *E. hormaechei* subsp. *hoffmannii* has been associated with reduced susceptibility to β-lactams and fluoroquinolones (51, 52). These findings suggest that, in regions where non-DTR CRE predominate, certain species or clones may warrant focused surveillance because of a possible association with DTR phenotypes. Given the small sample size, these observations should be considered exploratory.

This study has several limitations. First, because the analysis included only CRE isolates, we could not assess the overall proportion of DTR among all Enterobacterales infections. Second, the small number of DTR cases limited statistical power to detect outcome differences. This reflects the low incidence of CRE infections and the low frequency of DTR in IMP-predominant settings in Japan. Third, given the heterogeneity of pathogens, resistance mechanisms, infection syndromes, and treatment regimens, our analyses should be interpreted as exploratory and descriptive rather than as definitive assessments of treatment effectiveness. Fourth, newer agents such as cefiderocol and newer BL/BLI combinations were not available in Japan during the study period, and their potential therapeutic impact could not be evaluated. Finally, prior studies reporting associations between the DTR phenotype and clinical outcomes have often relied on large clinical databases (10, 11). Differences in study design, patient populations, and available clinical details may limit direct comparisons with our prospective multicenter cohort. These limitations underscore the need for further region-specific studies to clarify the clinical relevance of the DTR definition.

### Conclusion

This study demonstrates that non-DTR CRE predominate in Japan, consistent with the predominance of IMP-type carbapenemases and in contrast to regions where KPC, NDM, or VIM carbapenemases are more common. Our genomic analysis suggests that globally disseminated carbapenem-resistant clones are also circulating within the country. Clinical outcomes, including mortality and DOOR, were similar between patients with DTR CRE and those with non-DTR CRE infections, despite frequent mismatch between susceptibility and administered therapy. These findings highlight the need for continued regional multicenter studies to evaluate the treatment and outcomes of CRE infections and to assess the utility of DTR classification in Enterobacterales infections, particularly in settings with region-specific resistance epidemiology.

## Data Availability

The genome sequencing data reported in this study are available under the BioProject accession numbers PRJNA1040422 and PRJDB37866, which are listed in Table S5. Deidentified individual participant data that underlie the results reported in this article (including text, tables, figures, and appendices) will be made available upon reasonable request to the corresponding author, subject to approval of a methodologically sound proposal, execution of a data access agreement, and compliance with applicable institutional and ethical requirements.

## SUPPLEMENTAL MATERIAL

Supplemental material spectrum.01007-26-s0001.pdf Tables S1 to S4; Fig. S1 to S3.

Table S5 spectrum.01007-26-s0002.xlsx List of study isolates and corresponding accession numbers.

ASM does not own the copyrights to Supplemental Material that may be linked to, or accessed through, an article. The authors have granted ASM a non-exclusive, world-wide license to publish the Supplemental Material files. Please contact the corresponding author directly for reuse.
