# Supplementary material for: Difficult-to-treat resistance (DTR), treatment, and outcomes of carbapenem-resistant Enterobacterales infections in the setting of IMP-type carbapenemase predominance in Japan
Source: Microbiol Spectr. 2026 Apr 27;14(6):e01007-26. doi: 10.1128/spectrum.01007-26 (PMC13227980; doi:10.1128/spectrum.01007-26)
Supplement: Supplemental material — Tables S1 to S4; Fig. S1 to S3. [file spectrum.01007-26-s0001.pdf]

Supplementary Table 1. Component definitions for desirability of outcome ranking (DOOR) endpoint.

Component	Definition
Clinical failure	Did not meet clinical cure as assessed by the study investigator, or met any of the following criteria between the date of culture collection and the end of antimicrobial therapy:<br>• Death within 48 hours of culture collection<br>• Intensive care unit admission<br>• New requirement for mechanical ventilation<br>• New requirement for hemodialysis
Infectious complications	• Septic shock<br>• Cardiac arrest<br>• Empyema<br>• Renal abscess
Serious adverse events	• Acute kidney injury<br>• Allergy<br>• Other

Rank	Alive	How many of the following:<br>1. Absence of clinical response<br>2. Infectious complications<br>3. Serious adverse events
1	Yes	0 of 3
2	Yes	1 of 3
3	Yes	2 of 3
4	Yes	3 of 3
5	No (death)	Any

Note: Clinical cure was defined as resolution of infection-related symptoms and signs, including fever and focal symptoms. Clinical failure was classified hierarchically: the investigator's overall assessment was used when available. Otherwise, predefined objective components were applied.

Supplementary Table 2. Distribution of sequence types.

	All (n = 64)<br>n (%)
Enterobacter cloacae complex	21 (32.8)
ST 133	7
ST 78	3
ST 53	1
ST 113	1
ST 120	1
ST 175	1
ST 421	1
ST 484	1
ST 563	1
ST 693	1
ST 2795	1
ST 27 SLV	1
NT	1
Klebsiella aerogenes	8 (12.5)
ST 4	1
ST 30	1
ST 103	1
ST 227	1
ST 338	1
ST 383	1
ST 526	1
NT	1
Klebsiella pneumoniae complex	13 (20.3)
ST 12	1
ST 25	1
ST 35	1
ST 37	1
ST 290	1
ST 359	1
ST 517	1
ST 1320	1
ST 1691	1
ST 4459	1
ST 6808	1
ST 6809	1
ST 1026 SLV	1
Klebsiella oxytoca complex	8 (12.5)
ST 43	3
ST 27	1
ST 40	1
ST 176	1
ST 310	1
ST 43 SLV	1
Escherichia coli	3 (4.7)
ST 38	1
ST 131	1
ST 1290	1
Citrobacter spp.	4 (6.2)
ST 396	1

ST 1136	1
ST 497 SLV	1
NT	1
Proteus mirabilis	3 (4.7)
ST 133	1
ST 154	1
ST 162 SLV	1
Serratia spp.	4 (6.2)
ST 367	1
NT	3

Abbreviations: NT, non-typeable; SLV, single locus variant; ST, sequence type.

Supplementary Table 3. Distribution of desirability of outcome ranking (DOOR) components.

DOOR Components	All (n = 64)	DTR CRE (n = 12)	Non-DTR CRE (n = 52)	IMP-producing CRE (n = 30)
**Clinical failure**	26 (40.6)	4 (33.3)	22 (42.3)	12 (40.0)
Death within 48 hours of culture collection	1 (1.6)	0 (0.0)	1 (1.9)	1 (3.3)
Intensive care unit admission	17 (26.6)	3 (25.0)	14 (26.9)	7 (23.3)
New requirement for mechanical ventilation	9 (14.1)	1 (8.3)	8 (15.4)	4 (13.3)
New requirement for hemodialysis	2 (3.1)	0 (0.0)	2 (3.8)	1 (3.3)
**Infectious complications**	16 (25.0)	3 (25.0)	13 (25.0)	6 (20.0)
Septic shock	14 (21.9)	3 (25.0)	11 (21.2)	5 (16.7)
Cardiac arrest	3 (4.7)	1 (8.3)	2 (3.8)	3 (10.0)
Empyema	1 (1.6)	0 (0.0)	1 (1.9)	0 (0.0)
Renal abscess	0 (0.0)	0 (0.0)	0 (0.0)	0 (0.0)
**Serious adverse events**	2 (3.1)	0 (0.0)	2 (3.8)	0 (0.0)
Acute kidney injury	2 (3.1)	0 (0.0)	2 (3.8)	0 (0.0)
Allergy	1 (1.6)	0 (0.0)	1 (1.9)	0 (0.0)
Other	1 (1.6)	0 (0.0)	1 (1.9)	0 (0.0)

Note: Data are n (%). Other included thrombocytopenia. Component categories were not mutually exclusive, and a single patient could meet criteria for more than one component.
Abbreviations: CRE, carbapenem-resistant Enterobacterales; DOOR, desirability of outcome ranking; DTR, difficult-to-treat resistance.

Supplementary Table 4. Reference genomes.

Species	Taxonomy ID	NCBI RefSeq assembly	BioProject	BioSample ID	Strain
Citrobacter braakii	57706	GCF_009648935.1	PRJNA541977	SAMN10574724	MiY-A
Citrobacter europaeus	1914243	GCF_037482245.1	PRJNA1088829	SAMN40501937	WF1643
Citrobacter freundii	546	GCF_904859905.1	PRJNA646837	SAMEA3357462	MSB1_1H
Enterobacter asburiae	61645	GCF_007035805.1	PRJDB8163	SAMD00166968	17Nkhm-UP2
Enterobacter hormaechei	158836	GCF_019048245.1	PRJNA231221	SAMN16357575	FDAARGOS 1433
Enterobacter hormaechei subsp. *steigerwaltii*	299766	GCF_001729725.1	PRJNA259658	SAMN05581751	DSM 16691
Enterobacter hormaechei subsp. *hoffmannii*	1812934	GCF_001729745.1	PRJNA259658	SAMN05581748	DSM 14563
Enterobacter hormaechei subsp. *oharae*	301102	GCF_001729705.1	PRJNA259658	SAMN05581749	DSM 16687
Enterobacter hormaechei subsp. *xiangfangensis*	1296536	GCF_001729785.1	PRJNA259658	SAMN05581746	LMG27195
Enterobacter kobei	208224	GCF_023023125.1	PRJNA762162	SAMN21379554	11778-yvys
Enterobacter quasiroggenkampii	2497436	GCF_043972695.1	PRJNA1067818	SAMN39551828	ECC-072
Escherichia coli	511145	GCF_000005845.2	PRJNA225	SAMN02604091	K-12 substr. MG1655
Klebsiella aerogenes	548	GCF_019048125.1	PRJNA231221	SAMN16357584	FDAARGOS 1442
Klebsiella michiganensis	1134687	GCF_015139575.1	PRJDB9036	SAMD00196009	THO-011
Klebsiella oxytoca	571	GCF_900636985.1	PRJEB6403	SAMEA3923594	NCTC13727
Klebsiella pneumoniae subsp. *pneumoniae*	1125630	GCF_000240185.1	PRJNA78789	SAMN02602959	HS11286
Klebsiella quasipneumoniae	1463165	GCF_020099175.1	PRJNA231221	SAMN21218859	FDAARGOS_1503
Klebsiella quasipneumoniae subsp. *similipneumoniae*	1463164	GCF_002187935.3	PRJNA351846	SAMN05960914	G747
Klebsiella quasipneumoniae subsp. *quasipneumoniae*	1667327	GCF_020525925.1	PRJNA768294	SAMN22024800	01A030T
Klebsiella variicola subsp. *variicola*	2590157	GCF_020525545.1	PRJNA768294	SAMN22024815	F2R9T
Proteus mirabilis	529507	GCF_000069965.1	PRJNA12624	SAMEA1705945	HI4320
Serratia marcescens	615	GCF_030291735.1	PRJNA981493	SAMN35676739	ELP1.10
Serratia marcescens subsp. *marcescens*	911022	GCF_017654245.1	PRJNA716961	SAMN18473280	ATCC 13880
Serratia nevei	2703794	GCF_037948395.1	PRJNA1081831	SAMN40188831	LMG 31536
Serratia sarumanii	3020826	GCF_035749905.1	PRJNA1062297	SAMN39290590	K-M0228

Supplementary Figure 1. Forest plot demonstrating the desirability of outcome ranking (DOOR).

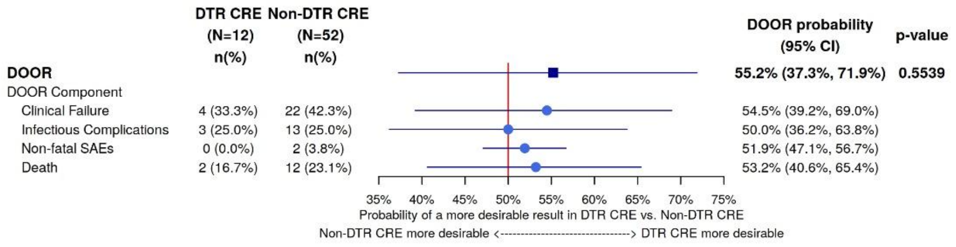

Abbreviations: CI, confidence interval; CRE, carbapenem-resistant Enterobacterales; DTR, difficult-to-treat resistance; SAEs, serious adverse events

Supplementary Figure 2. Kaplan-Meier survival curves by carbapenemase group.

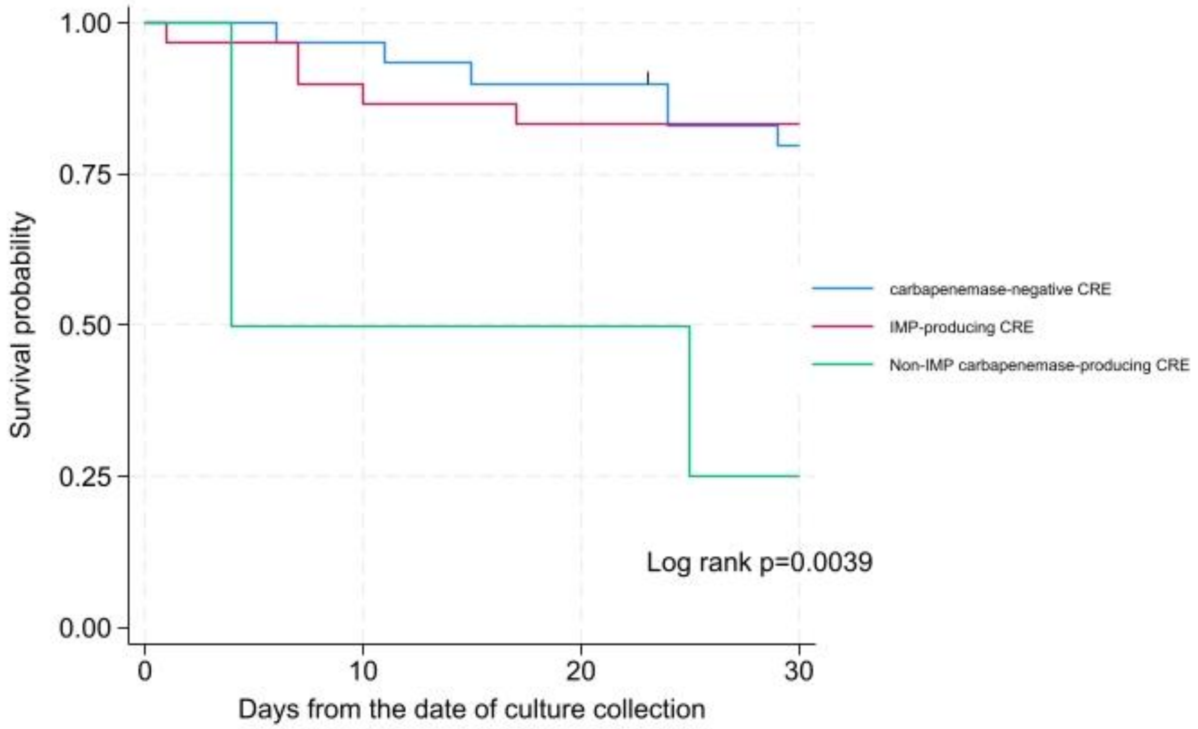

Abbreviations: CRE, carbapenem-resistant Enterobacterales.

Supplementary Figure 3. Exploratory desirability of outcome ranking (DOOR) distributions by carbapenemase group.

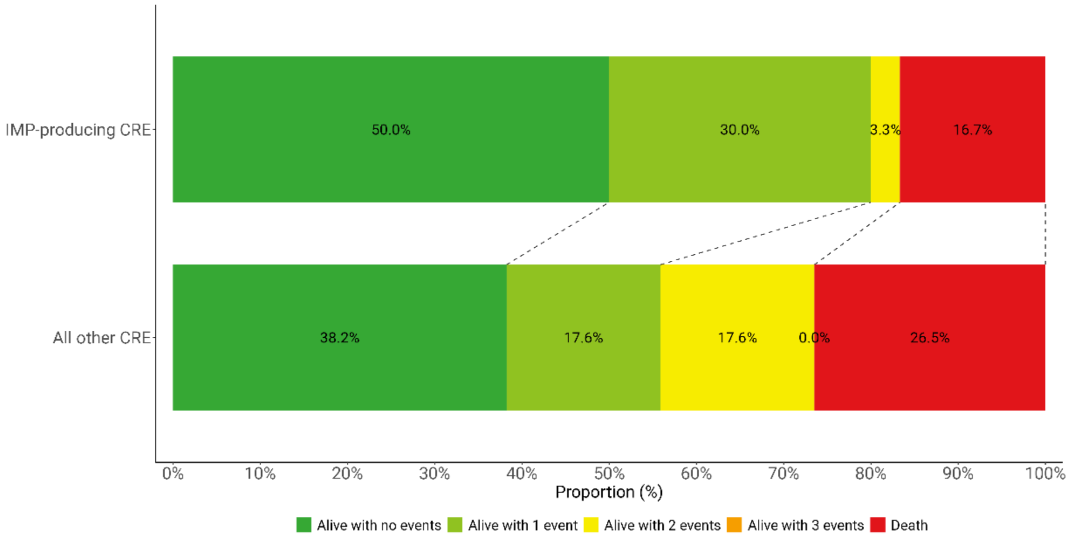

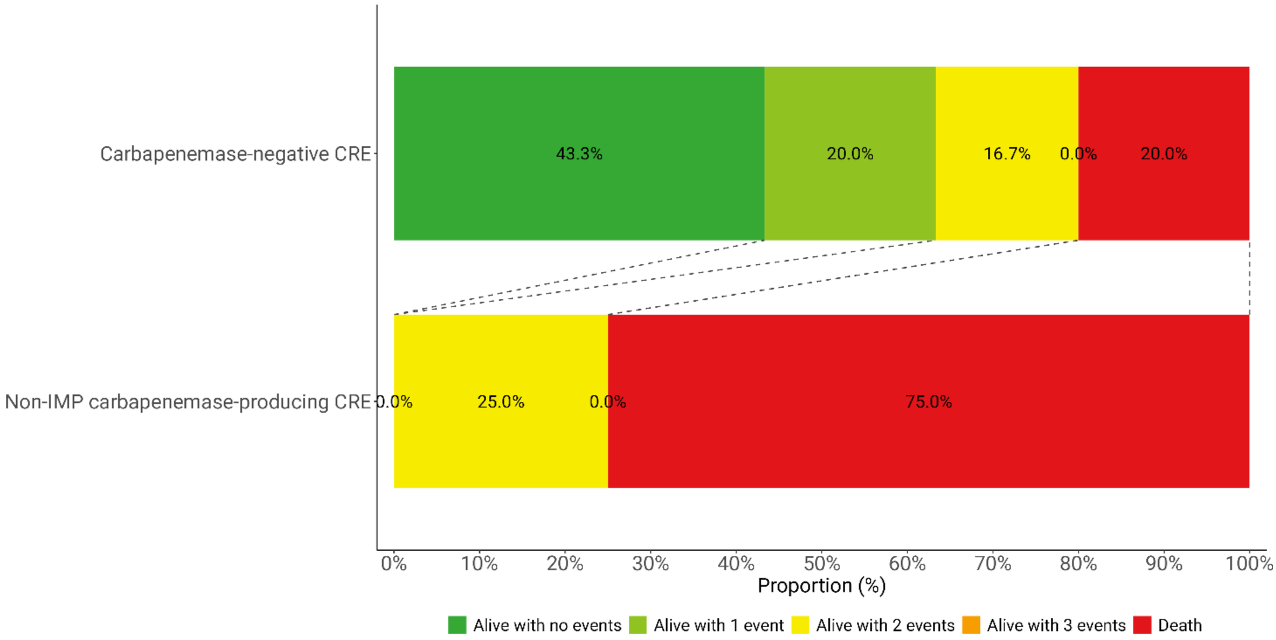

Note: The upper panel compares IMP-producing CRE with all other CRE. The lower panel further subdivides the non-IMP group into non-IMP carbapenemase-producing CRE and carbapenemase-negative CRE.

Abbreviations: CRE, carbapenem-resistant Enterobacterales; DOOR, desirability of outcome ranking.
